# Neuroprotection and spatial memory enhancement of four herbal mixture extract in HT22 hippocampal cells and a mouse model of focal cerebral ischemia

**DOI:** 10.1186/s12906-015-0741-1

**Published:** 2015-06-30

**Authors:** Sung Min Ahn, Yu Ri Kim, Ha Neui Kim, Young Whan Choi, Jae Won Lee, Cheol Min Kim, Jin Ung Baek, Hwa Kyoung Shin, Byung Tae Choi

**Affiliations:** Department of Korean Medical Science, School of Korean Medicine, Pusan National University, Yangsan, 626-870 Republic of Korea; Department of Horticultural Bioscience, College of Natural Resource and Life Science, Pusan National University, Miryang, 626-706 Republic of Korea; Division of Pharmacy, College of Pharmacy, Pusan National University, Busan, 609-735 Republic of Korea; Department of Biochemistry, College of Medicine, Pusan National University, Yangsan, 626-870 Republic of Korea; Division of Humanities and Social Medicine, School of Korean Medicine, Pusan National University, Yangsan, 626-870 Republic of Korea; Division of Meridian and Structural Medicine, School of Korean Medicine, Pusan National University, Yangsan, 626-870 Republic of Korea; Research Center for Anti-aging Technology Development, Pusan National University, Busan, 609-735 Republic of Korea

**Keywords:** Hippocampal cell, Neuroprotection, Memory, *Polygonum multiflorum*

## Abstract

**Background:**

Four traditional Korean medicinal herbs which act in retarding the aging process, *Polygonum multiflorum* Thunb*.*, *Rehmannia glutinosa* (Gaertn) Libosch*.*, *Polygala tenuifolia* Willd., and *Acorus gramineus* Soland., were prepared by systematic investigation of Dongeuibogam (Treasured Mirror of Eastern Medicine), published in the early 17th century in Korea. This study was performed to evaluate beneficial effects of four herbal mixture extract (PMC-12) on hippocampal neuron and spatial memory.

**Methods:**

High performance liquid chromatography (HPLC) analysis was performed for standardization of PMC-12. Cell viability, lactate dehydrogenase, flow cytometry, reactive oxygen species (ROS), and Western blot assays were performed in HT22 hippocampal cells and immunohistochemistry and behavioral tests were performed in a mouse model of focal cerebral ischemia in order to observe alterations of hippocampal cell survival and subsequent memory function.

**Results:**

In the HPLC analysis, PMC-12 was standardized to contain 3.09 % 2,3,5,4′-tetrahydroxystilbene-2-O-β-D-glucoside, 0.35 % 3′,6-disinapoyl sucrose, and 0.79 % catalpol. In HT22 cells, pretreatment with PMC-12 resulted in significantly reduced glutamate-induced apoptotic cell death. Pretreatment with PMC-12 also resulted in suppression of ROS accumulation in connection with cellular Ca^2+^ level after exposure to glutamate. Expression levels of phosphorylated p38 mitogen-activated protein kinases (MAPK) and dephosphorylated phosphatidylinositol-3 kinase (PI3K) by glutamate exposure were recovered by pretreatment with either PMC-12 or anti-oxidant N-acetyl-L-cysteine (NAC). Expression levels of mature brain-derived neurotrophic factor (BDNF) and phosphorylated cAMP response element binding protein (CREB) were significantly enhanced by treatment with either PMC-12 or NAC. Combination treatment with PMC-12, NAC, and intracellular Ca^2+^ inhibitor BAPTA showed similar expression levels. In a mouse model of focal cerebral ischemia, we observed higher expression of mature BDNF and phosphorylation of CREB in the hippocampus and further confirmed improved spatial memory by treatment with PMC-12.

**Conclusions:**

Our results suggest that PMC-12 mainly exerted protective effects on hippocampal neurons through suppression of Ca^2+^-related ROS accumulation and regulation of signaling pathways of p38 MAPK and PI3K associated with mature BDNF expression and CREB phosphorylation and subsequently enhanced spatial memory.

## Background

Due to an increase in life expectancy and the elderly population, memory and cognitive impairments including dementia have become a major public health problem [1]. Hippocampal neuronal death is a major factor in the progress of memory impairment in many brain disorders [2, 3]. Prevention of hippocampal neuronal deaths provides a potential new therapeutic strategy to ameliorate memory and cognitive impairment of many neurological disorders. HT22 hippocampal cell line, which lacks a functional glutamate receptor, is valuable for studying molecular mechanism of memory deficits [2, 4].

Exposure of HT22 hippocampal cells to glutamate shows neurotoxicity through oxidative stress rather than N-methyl-D-aspartate receptor-mediated excitotoxicity [5–7]. Non-receptor-mediated oxidative stress involves inhibition of cysteine uptake, alterations of intracellular cysteine homeostasis, glutathione depletion, and ultimately elevation of reactive oxygen species (ROS) activation inducing neuronal cell death [5, 8–10]. Death of hippocampal cell following oxidative stress and accumulation of ROS play a role in learning and memory impairment of brain disorders [11].

Under oxidative neuronal death, the pathways of p38 mitogen-activated protein kinase (MAPK) and phosphatidylinositol-3 kinase (PI3K) play critical roles in control of neuronal death and cell survival caused by glutamate, respectively [6, 12]. Brain-derived neurotrophic factor (BDNF) mediates neuronal survival and neuroplasticity and associated learning and memory, and its signaling is associated with alteration of the main mediators of PI3K pathways [13–15]. Cyclic AMP response element binding protein (CREB) has multiple roles in different brain areas, as well as promotion of cell survival [16, 17] and is involved in memory, learning, and synaptic transmission in the brain [18, 19].

Studies of the neuroprotection have been performed in both HT22 cells and middle cerebral artery occlusion (MCAO)-induced injury [20, 21]. However memory deficits are also frequently noted after stroke. Transient MCAO induce a progressive deficiency in spatial performance related to impaired hippocampal function [22]. Thus shorter durations of ischemia have been used in the experiments that aimed to test impaired spatial learning and memory performance [23].

In traditional literature of Korean/Chinese medicine, research for screening, evaluating citation and attempting practical use of herbs has been conducted for development of therapeutic strategies [24]. We prepared multiherb formulae comprising the roots of *Polygonum multiflorum*, *Rehmannia glutinosa*, *Polygala tenuifolia*, and *Acorus gramineus* to increase medicinal efficacy by systematic investigation of Dongeuibogam, published by Joon Hur in the early 17th century in Korea. Our aim was to determine the beneficial effects of herbal mixture extract on hippocampal neurons, a susceptible cell important in memory impairment, in HT22 hippocampal cells and the hippocampus with subsequent memory enhancement in a mouse model of focal cerebral ischemia.

## Methods

### Chemicals and antibodies

L-glutamate, 3-(4,5-dimethylthiazol-2-yl)-2,5-diphenyltetrazolium bromide (MTT), N-acetyl-L-cysteine (NAC), and β-actin antibody were purchased from Sigma-Aldrich (St. Louis, MO, USA). BAPTA-AM and EGTA were purchased from Tocris Bioscience (Ellisville, MO, USA). Dulbecco’s modified Eagle’s medium (DMEM), fetal bovine serum (FBS), and other cell culture reagents were purchased from Gibco-Invitrogen (Carlsbad, CA, USA). Antibodies recognizing p38, phospho-p38 (pp38, Thr180/Tyr182), PI3K, and pro-BDNF were supplied by Santa Cruz Biotechnology (Santa Cruz, CA, USA), and CREB, phospho-CREB (pCREB, Ser133), and phospho-PI3K (pPI3K, Tyr458) were supplied by Cell Signaling (Danvers, MA, USA). Antibody recognizing neuronal nuclei (NeuN) was supplied by Millipore Corporation (Billerica, MA, USA), and mature BDNF was supplied by Abcam (Cambridge, MA, USA). Secondary antibodies were supplied by Santa Cruz Biotechnology. A FITC Annexin V apoptosis detection kit was purchased from BD Bioscience (San Diego, CA, USA). A lactate dehydrogenase (LDH) cytotoxicity assay kit was purchased from Promega (Madison, WI, USA), and ROS detection reagent, 5-(and-6)-carboxy-2′,7′-dichlorodihydrofluorescein diacetate (carboxy-H_2_DCFDA), and Hoechst 33342 was purchased from Invitrogen (Carlsbad, CA, USA).

### Preparation of four herbal mixture extract

The dried roots of *Polygonum multiflorum* Thunb*.*, *Rehmannia glutinosa* (Gaertn) Libosch*.*, *Polygala tenuifolia* Willd., and *Acorus gramineus* Soland. were purchased from Dongnam Co. (Busan, Korea) and were authenticated by Professor Y.W. Choi, Department of Horticultural Bioscience, College of Natural Resource and Life Science, Pusan National University. A voucher specimen (accession number PMCWSD2.1 ~ 2.4) was deposited at the Plant Drug Research Laboratory of Pusan National University (Miryang, Korea). Dried powdered *Polygonum multiflorum* (25.5 kg), *Rehmannia glutinosa* (9.5 kg), *Polygala tenuifolia* (7.5 kg), and *Acorus gramineus* roots (7.5 kg) were immersed in 450 L of distilled water and boiled at 120 ± 5 °C for 150 min. The resultant extract was centrifuged (2000 × *g* for 20 min at 4 °C) and filtered through a 0.2-μm filter. The filtrate was then concentrated in vacuo at 70 ± 5 °C under reduced pressure and then converted into a fine spray-dried powder at a yielding rate of 4.6 % (2.3 kg) in a vacuum drying apparatus. Finally, the solid form of the spray-dried powder was dissolved with dimethyl sulfoxide (DMSO) for use as PMC-12 in experiments.

### High performance liquid chromatography (HPLC) analysis and quantification

For analysis of quality and quantity for PMC-12, sample of 0.5 g dry weight was sonicated in 10 ml MeOH, filtered through a 0.45 μm membrane filter before HPLC analysis. HPLC using G1100 systems (Agilent Technologies, Waldbronn, Germany) was performed on a Luna C_18_ column (5 μm, 150 mm × 3.0 mm i.d. Phenomenex, Torrance, CA, USA) with a mobile phase gradient of acetonitrile–water (0 to 100) for 35 min. The injection volume was 10 μl of sample and mobile phase flow rate 0.4 ml/min with UV detection at 254 nm for 2,3,5,4′-tetrahydroxystilbene-2-O-β-D-glucoside (THS) and 3′,6-disinapoyl sucrose (DISS) and at 203 nm for catalpol. Acquisition and analysis of chromatographic data were performed using Agilent chromatographic Work Station software (Agilent Technologies). Stock solutions of THS, DISS, and catalpol were prepared for quantification of PMC-12. The contents of PMC-12 were determined by regression equations, calculated in the form of y = ax + b, where x and y were peak area and contents of the compound. The limits of detection (LOD) and quantification (LOQ) under the current chromatographic conditions were determined at a signal-to-noise ratio of 3 and 10, respectively.

### Cell culture

HT22 cells were cultured in DMEM supplemented with 10 % FBS and 1 % penicillin/streptomycin in a 5 % CO_2_ humidified incubator at 37 °C. The cells were incubated for 24 h prior to experimental treatments. After incubation, cells were treated with various concentrations of PMC-12 for 24 h, followed by exposure to 5 mM glutamate for 24 h. Cells were pretreated with inhibitors for 30 min before addition of PMC-12 and then exposed to glutamate.

### Cell viability assay

HT22 cell survival was assessed using a MTT assay. The culture medium was replaced with 0.5 mg/ml MTT solution and then left in a dark place for 4 h at 37 °C. Following incubation, the cells were treated with DMSO in order to dissolve the formazan crystals. Absorbance was determined at 595 nm using a SpectraMax 190 spectrophotometer (Molecular Devices, Sunnyvale, CA, USA). Results were expressed as a percentage of control.

### Determination of cell cytotoxicity

Released LDH from damaged cells was measured for estimation of cytotoxicity. For induction of maximal cell lysis, treated cells were lysed with 0.9 % Triton X-100 for 45 min at 37 °C. Supernatant samples were transferred to a 96-well enzymatic assay plate and reacted with substrate mix in the dark for 30 min at room temperature. At the end of that time, samples were treated with stop solution and read at 490 nm using a SpectraMax 190 spectrophotometer (Molecular Devices). Data represent the percentage of LDH released relative to controls.

### Flow cytometric analysis

After treatment, cells were harvested and resuspended in binding buffer at a concentration of 1x10^4^ cells/ml. For analysis of cell death types, 100 μl of the solution was transferred to a flow cytometric tube, followed by incubation with Annexin V-FITC and propidium iodide (PI) in the dark at room temperature for 15 min. Subsequently, 400 μl of binding buffer was added and analysis of the samples was performed using a flow cytometer (FACS Canto™ II; Becton-Dickinson, San Jose, CA, USA).

### ROS measurement

HT22 cells were cultured in 96-well white plates at a density of 5x10^3^ cells per well. After adherence, cells were pretreated with PMC-12 for 24 h and then exposed to 5 mM glutamate for 24 h. Treated cells were washed with PBS. Carboxy-H_2_DCFDA (20 μM) (Invitrogen) was applied to the cells, followed by incubation for 1 h in a 37 °C incubator. Fluorescence was measured using a Mutilabel counter (Perkin Elmer 1420, MA, USA). Accumulation of intracellular ROS was observed and photographed under a fluorescence microscope (Carl Zeiss Imager M1, Carl Zeiss, Inc., Gottingen, Germany). In addition, cells were harvested, resuspended in 1 ml PBS with 20 μM carboxy-H_2_DCFDA (Invitrogen), and then incubated for 1 h at 37 °C. After washing, cellular fluorescence was measured using a flow cytometer.

### Nuclear staining with Hoechst 33342

Apoptosis was investigated by staining the cells with Hoechst 33342 (Invitrogen). HT22 cells were washed three times with PBS and then fixed in PBS containing 4 % paraformaldehyde for 25 min at 4 °C. Fixed cells were washed with PBS and stained with Hoechst 33342 (10 μg/ml) for 15 min at room temperature. The cells were washed three times with PBS and mounted using the medium for fluorescence (Vector Laboratories, Inc.) The cells were observed under a fluorescence microscope for nuclei showing typical apoptotic features such as chromatin condensation and fragmentation. Photographs were taken at a magnification of X 200.

### Western blot analysis

The cells were homogenized with lysis buffer [200 mM Tris (pH 8.0), 150 mM NaCl, 2 mM EDTA, 1 mM NaF, 1 % NP40, 1 mM PMSF, 1 mM Na_3_VO_4_, protease inhibitor cocktail]. Equal amounts of proteins were separated by 10 or 12 % sodium dodecyl sulfate-polyacrylamide gel electrophoresis (SDS-PAGE) and then transferred to a nitrocellulose membrane (Whatman GmbH, Dassel, Germany). The membranes were blocked with 5 % skim milk in PBST for 1 h, followed by overnight exposure to appropriate antibodies. Membranes were then incubated with appropriate horseradish peroxidase-conjugated antibodies for 1 h. All specific bands were visualized using an enhanced chemiluminescence system (Pierce Biotech, Rockford, IL, USA) and imaged using an Image Quant LAS-4000 imaging system (GE Healthcare Life Science, Uppsala, Sweden). Results of the Western blot assay reported here are representative of at least three experiments.

### Focal cerebral ischemia

To confirm beneficial effects of PMC-12 on hippocampal cell, we used middle cerebral artery occlusion (MCAO) model. Male C57BL/6 mice (20-25 g) were obtained from Dooyeol Biotech (Seoul, Korea). The mice were housed at 22 °C under alternating 12 h cycles of dark and light, and were fed a commercial diet and allowed tap water *ad libitum*. All experiments were approved by the Pusan National University Animal Care and Use Committee. Each group consisted of six mice and all treatments were administered under isoflurane (Choongwae, Seoul, Korea) anesthesia, which was provided using a calibrated vaporizer (Midmark VIP3000, Orchad Park, OH, USA).

Focal cerebral ischemia was induced by occluding the middle cerebral artery (MCA) using the intraluminal filament technique. A fiber-optic probe was affixed to the skull over the middle cerebral artery for measurement of regional cerebral blood flow using a PeriFlux Laser Doppler System 5000 (Perimed, Stockholm, Sweden). Middle cerebral artery occlusion model was induced by a silicon-coated 4-0 monofilament in the internal carotid artery and the monofilament was advanced to occlude the MCA. The filament was withdrawn 30 min after occlusion and reperfusion was confirmed using laser Doppler. Mice in the PMC-12 groups received oral administration daily at the doses of 100 and 500 mg/kg for three weeks after MCAO, while mice in the control and vehicle groups were only given distilled water at the same intervals.

### Immunofluorescence staining

Mice anesthetized with isoflurane received intracardial perfusion with saline and then 4 % paraformaldehyde in PBS. Brains were removed, post-fixed in the same fixative for 4 h at 4 °C, and immersed in 30 % sucrose for 48 h at 4 °C for cryoprotection. Frozen 20 μm-thick sections were incubated for blocking with a blocking buffer (1X PBS/5 % normal serum/0.3 % Triton X-100) for 1 h at room temperature. The sections were incubated with the following primary antibodies to NeuN (Millipore Corporation), mature BDNF (Abcam), and pCREB (Cell Signaling) overnight in PBS at 4 °C. After washes with PBS, the sections were incubated with the fluorescent secondary antibody (Vector Laboratories, Inc., Burlingame, CA, USA) at room temperature in the dark, respectively, and then washed three times with PBS. Subsequently, slides were mounted in the mounting medium (Vector Laboratories, Inc.) and captured using a fluorescence microscope.

### Behavioral assessment

Acquisition training for the Morris water maze was performed on four consecutive days from 10 days to seven days before MCAO (five trials per day) and basal time was measured at six days before MCAO. The tank had a diameter of 100 cm and an altitude of 50 cm. The platform was placed 0.5 cm beneath the surface of the water. Each trial was performed for 90 s or until the mouse arrived on the platform. PMC-12-treated mice received daily oral administration at the doses of 100 and 500 mg/kg for three weeks after MCAO, while mice in the control and vehicle groups were only given distilled water at the same intervals. After final administration, results of the experiment were recorded using SMART 2.5.18 (Panlab S.L.U.).

### Data analysis

All data were expressed as mean ± SEM and analyzed using the SigmaStat statistical program version 11.2 (Systat Software, San Jose, CA, USA). Statistical comparisons were performed using one-way analysis of variance (ANOVA) for repeated measures followed by Tukey’s test of least significant difference. A *P*-value < 0.05 was considered to indicate a statistically significant result. The median effective dose (ED_50_) value of PMC-12 (*in vitro* experiments) was derived from dose–response curve.

## Results

### HPLC analysis of PMC-12

**Fig. 1:**
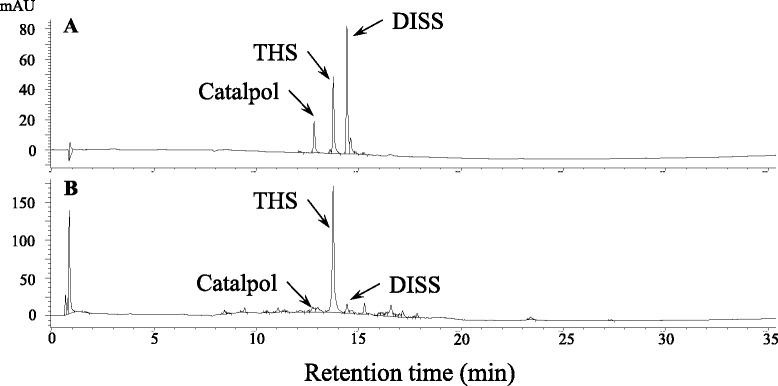
HPLC analysis and quantification of PMC-12. HPLC chromatograms of THS, DISS, and catalpol reference (**a**) and PMC-12 (**b**) obtained using a Luna C18 (2) column monitored at 254 nm and eluted with 100 % water to 100 % acetonitrile for 40 at a flow-rate of 1.0 ml/min

HPLC conditions, particularly the mobile phase and its elution program, are important for determination of the compound in the biological matrix. In this study, we found that a mobile phase consisting of acetonitrile and containing H_2_O can separate THS, DISS, and catalpol (Fig. 1). The HPLC conditions developed in this study produced full peak-to-baseline resolution of the major active THS, DISS, and catalpol present in PMC-12. Based on UV maximal absorption, we detected THS and DISS at 254 nm and catalpol at 203 nm for quantitative analysis. The contents of THS, DISS, and catalpol in PMC-12 were 3.085 ± 0.271 %, 0.352 ± 0.058 %, and 0.785 ± 0.059 %, respectively. Linear calibration curve showed good linear regression (r^2^ > 0.999) within test ranges; the LOD (S/N = 3) and the LOQ (S/N = 10) were less than 1.5 and 4.5 μg at 254 nm for THS and DISS and at 203 nm for the catalpol (Table 1).

**Table 1:** Concentration, calibration curve, regression data, LODs and LOQs for THS, DISS, and catalpol by HPLC

Compounds	Wavelength (nm)	Concentration (%)	Calibration curve	r^2^	LOD (μg/ml)	LOQ (μg/ml)
THS	254	3.085 ± 0.271	y = 3284.72x + 2.00	0.999	1.41	4.27
DISS	254	0.352 ± 0.058	y = 4289.06x + 47.95	0.999	0.59	1.79
Catalpol	203	0.785 ± 0.059	y = 1253.49x + 31.37	0.999	0.63	1.91

### Pretreatment with PMC-12 reduces glutamate-induced neuronal toxicity in HT22 cells

**Fig. 2:**
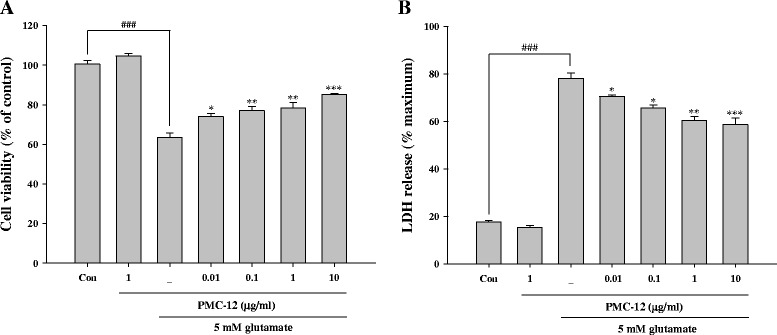
Protective effects of PMC-12 against glutamate-induced cell death in HT22 cells. Cell viability and toxicity were determined by MTT (**a**) and LDH assay (**b**). Cells were pretreated with 0.01, 0.1, 1, and 10 μg/ml of PMC-12 for 24 h, followed by exposure to 5 mM glutamate for 24 h. ^###^ *P* < 0.001 vs. control; *P < 0.05, **P < 0.01 and ***P < 0.001 vs. glutamate-treated cells. All data are represented as the mean ± SEM of three independent experiments

Exposure of cells to glutamate resulted in reduced cell viability of approximately 36.5 % compared with the control. Pretreatment with PMC-12 at a concentration range of 0.01 to 10 μg/ml (ED_50_ = 0.32 μg/ml PMC-12) resulted in significantly reduced glutamate-induced cytotoxicity in a dose-dependent manner (Fig. 2a). The levels of LDH release showed a significant increase to 77.8 % after exposure to glutamate, while pretreatment with PMC-12 resulted in a marked decrease of glutamate-induced release of LDH (Fig. 2b). These results suggest that pretreatment with PMC-12 exerts a potent neuroprotective effect against oxidative toxicity caused by exposure of HT22 cells to glutamate.

### Pretreatment with PMC-12 inhibits glutamate-induced apoptotic cell death in HT22 cells

**Fig. 3:**
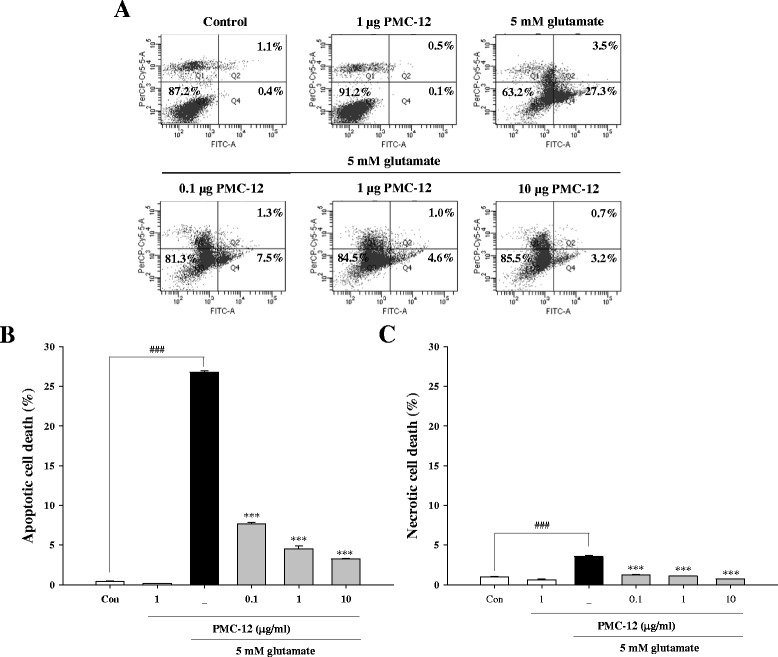
Protective effect of PMC-12 on types of cell death in HT22 cells. Cells were pretreated with 0.1, 1, or 10 μg/ml PMC-12 for 24 h, followed by exposure to 5 mM glutamate for 24 h. Representative flow cytometric analysis scatter-grams of Annexin V/PI staining (**a**) and quantitative analysis of the histograms (**b** and **c**). ^###^ *P* < 0.001 vs. control; ***P < 0.001 vs. glutamate-treated cells. All data are represented as the mean ± SEM of three independent experiments

We performed flow cytometry analysis using Annexin V/PI staining in order to characterize the types of neuronal death. Concentrations of 0.1, 1, and 10 μg/ml of PMC-12 were selected for testing. After exposure to glutamate, cells were likely to undergo apoptotic cell death rather than necrotic death, however, pretreatment with PMC-12 resulted in a marked decrease in the apoptotic population (Fig. 3). These results suggest that pretreatment with PMC-12 exerts a neuroprotective effect through inhibition of glutamate-induced apoptotic cell death.

### Pretreatment with PMC-12 inhibits glutamate-induced production of ROS in HT22 cells

**Fig. 4:**
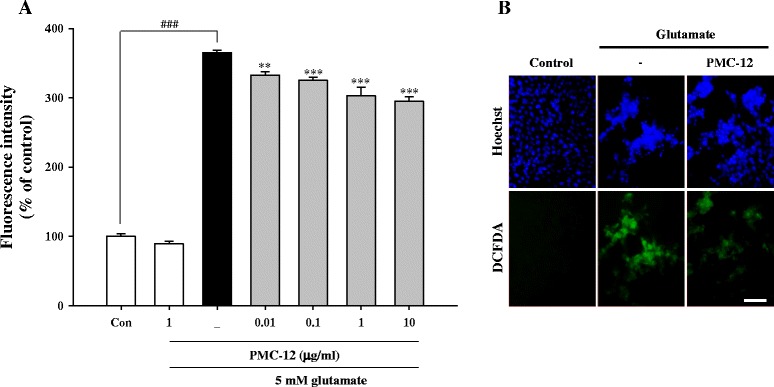
Protective effect of PMC-12 on ROS generation in glutamate-treated HT22 cells. Cells were pretreated with 0.01, 0.1, 1, or 10 μg/ml of PMC-12 for 24 h, followed by exposure to 5 mM glutamate for 24 h. The oxidation sensitive fluorescence dye, carboxy-H_2_DCFDA (20 μM), was used in measurement of ROS levels. Production of ROS was analyzed using a fluorescence plate reader (**a**) and fluorescence microscope (**b**). In addition, apoptotic nuclei were observed after staining with Hoechst 33342 for detection of apoptosis morphologically (**b**). ^###^ *P* < 0.001 vs. control; **P < 0.01 and ***P < 0.001 vs. glutamate-treated cells. All data are represented as the mean ± SEM of three independent experiments. Scale bars = 50 μm

**Fig. 5:**
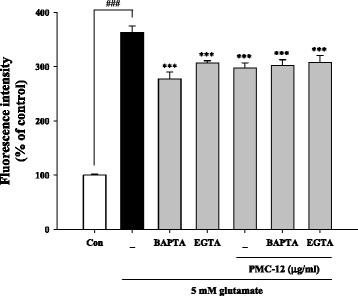
Protective effects of PMC-12 on cellular Ca^2+^-related ROS production in HT22 cells. Cells were pretreated with 10 μg/ml of PMC-12 for 24 h, followed by exposure to 5 mM glutamate for 24 h. Cells were treated with the specific Ca^2+^ inhibitors, 1.5 mM EGTA or 10 μM BAPTA-AM, for 30 min before addition of PMC-12 or glutamate. ROS production was measured using a fluorescence plate reader and mean fluorescence intensity was expressed as the mean ± SEM of three independent experiments. ^###^ *P* < 0.001 vs. control; ***P < 0.001 vs. glutamate-treated cells

Treatment of HT22 cells with glutamate resulted in increased production of ROS. However, pretreatment with PMC-12 resulted in a significant decrease in ROS production, which prevented elevation of ROS level caused by exposure to glutamate (Fig. 4a). We also performed staining with Hoechst 33342 to confirm morphological changes by glutamate-induced oxidative toxicity. Our result showed that PMC-12 protected against apoptotic cell death by production of ROS after exposure to glutamate (Fig. 4b). When cells were treated with 10 μM of intracellular Ca^2+^ chelator BAPTA-AM and 1.5 mM of extracellular Ca^2+^ chelator EGTA, both inhibitors caused a significant reduction in the levels of glutamate-induced production of ROS. However, no change in ROS production was observed in cells treated with a combination of Ca^2+^ chelators and PMC-12 compared to cells treated with PMC-12 alone followed by exposure to glutamate (Fig. 5). These results suggest that PMC-12 suppresses glutamate-induced oxidative stress by blocking ROS production, which may be related to an indirect Ca^2+^ pathway.

### Pretreatment with PMC-12 enhances mature BDNF expression and CREB phosphorylation via p38 MAPK and PI3K in HT22 cells

**Fig. 6:**
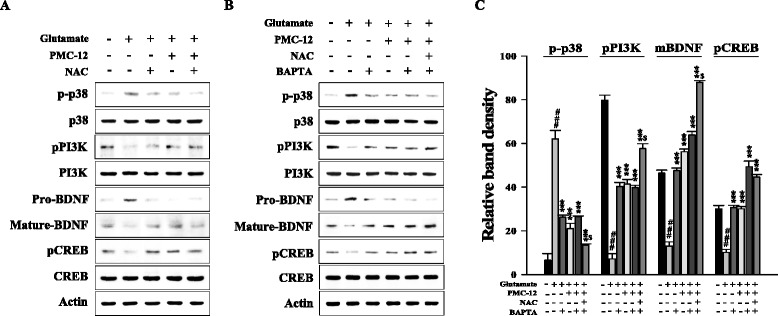
Protective effects of PMC-12 on regulation of intracellular protein kinases in HT22 cells. Cells were pretreated with 1 μg/ml PMC-12 or 1 mM NAC, followed by exposure to 5 mM glutamate (**a**). In addition, cells were pretreated with 10 μM BAPTA-AM or NAC for 30 min before addition of PMC-12 or glutamate (**b**). The histogram for Fig. 6b was indicated as the mean ± SEM of three independent experiments (**c**). Equal amounts of proteins and each sample were subjected to Western blot analysis using the indicated antibodies. Equal protein loading was confirmed by actin expression. ^###^ *P* < 0.001 vs. normal control^;^ ***P < 0.001 vs. glutamate-treated groups; ^$^ *P* < 0.05 vs. groups except control and glutamate-treated groups

Levels of phosphorylated p38 MAPK and dephosphorylated PI3K were significantly decreased by treatment with either PMC-12 or anti-oxidant NAC compared to glutamate-treated cells. Protein levels of mature BDNF and CREB phosphorylation were significantly increased by treatment with either PMC-12 or NAC (Fig. 6a). When cells were treated with PMC-12, NAC, or intracellular Ca^2+^ inhibitor BAPTA-AM, combination treatment resulted in markedly reduced levels of phosphorylated p38 MAPK and dephosphorylated PI3K compared to other cells. The combination treatment of cells also resulted in elevation of decreased protein levels of mature BDNF and CREB phosphorylation after exposure to glutamate (Fig. 6b). These results suggest that neuroprotective effects of PMC-12 may be regulated by both p38 MAPK and PI3K signaling with mature BDNF expression and CREB phosphorylation, and these effects may be related to ROS accumulation and Ca^2+^ influx.

### Treatment with PMC-12 enhances mature BDNF expression and CREB phosphorylation in the hippocampus of MCAO mice

**Fig. 7:**
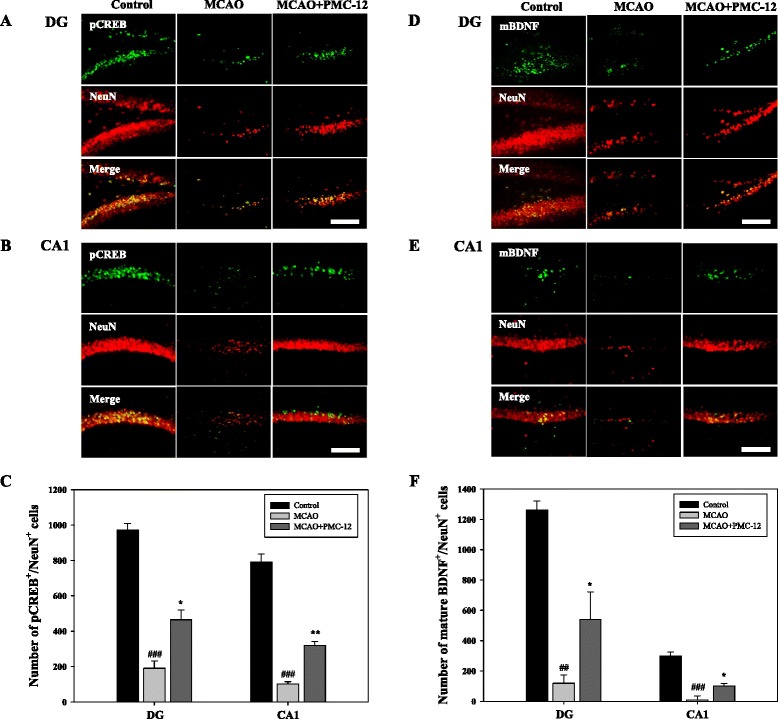
Protective effects of PMC-12 on expression of both pCREB and mature BDNF in the hippocampus of focal cerebral ischemia in mice at 26 days after MCAO. Photomicrograph (**a**-**b**, **d**-**e**) and its histogram for pCREB/NeuN (**c**) and mature BDNF/NeuN double-positive cells (**f**) in the DG and CA1 regions of the hippocampus. Total number of pCREB/NeuN and mBDNF/NeuN double-positive cells was significantly increased by administration of PMC-12 in DG and CA1 compared to the MCAO group. ^##^ *P* < 0.01 and ^###^ *P* < 0.001 vs. control^;^ *P < 0.05 and **P < 0.01 vs. MCAO mice. Scale bar = 100 μm

At 26 days after MCAO, positive neuronal cells of pCREB and mature BDNF in the hippocampal CA1 and dentate gyrus (DG) region were counted after immunofluorescence staining. Post-treatment with PMC-12 followed by MCAO surgery resulted in a significant increase in the number of double-positive cells of pCREB/NeuN or mature BDNF/NeuN in the CA1 and DG region of the ipsilateral hippocampus compared to the MCAO group (Fig. 7). These results suggest a possible association of the protective effects of PMC-12 with neuronal mature BDNF expression and CREB phosphorylation in the hippocampus of MCAO mice.

### Treatment with PMC-12 ameliorates spatial memory impairment in MCAO mice

**Fig. 8:**
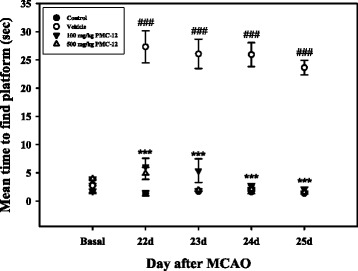
Beneficial effects of PMC-12 on the spatial memory function in MCAO mice. Morris water maze test was performed from 22 d to 25 d after MCAO. Administration of PMC-12 resulted in significantly improved memory function of MCAO mice during the late phase of the experiment. There was no significant differences in the results by two different doses (100 and 500 mg/kg) of PMC-12. Mean ± SEM. ^###^ *P* < 0.001 vs. control; ***P < 0.001 vs. MCAO mice (vehicle)

Spatial memory was assessed using the water maze test. MCAO mice took a longer time on average to find the platform than the basal group. However, PMC-12-administered mice attained a significantly lower time at both concentrations from 22 to 25 days after MCAO compared to the vehicle group (Fig. 8). In particular, it has shown that a low dose (100 mg/kg) of PMC-12 is enough for improvement of the damaged memory function. These results suggest that treatment with PMC-12 may induce beneficial effects for improvement of memory function in a focal cerebral ischemia model.

## Discussion

Traditional medical literature can be used to identify and shortlist herbs and combinations of herbs for further experimental and clinical research [24]. We have used a systematic method for screening and evaluating citations from the traditional Korean medicine literature, Dongeuibogam. In a screening exercise performed using HT22 hippocampal cells for selection of functional herbs on memory impairment, among many herbal candidates, we found that *Polygonum multiflorum* exhibited prominent neuroprotective effects.

Our previous results demonstrated that extracts from *Polygonum multiflorum* exert significant anti-apoptotic effects against glutamate-induced neurotoxicity [25, 26] and protect against cerebral ischemic damage via regulation of endothelial nitric oxide [27]. However, extracts of *Polygonum multiflorum* alone had a partial blocking effect against neuronal damage.

To enhance the potential beneficial effects of *Polygonum multiflorum*, we analyzed 4,014 herbal prescriptions mentioned in Dongeuibogam and noted 19 multiherb formulae, including the roots of *Rehmannia glutinosa*, *Polygala tenuifolia*, and *Acorus gramineus*. These 19 multiherbs are commonly used in treatment of mental and physical weakness of the elderly.

We prepared PMC-12 with *Polygonum multiflorum* base and above multiherb formulae to enhance medical efficiency. In a further screening exercise performed using a focal cerebral ischemia model, PMC-12 had marked effects on the decline of infarct size compared with *Polygonum multiflorum* extract alone. Thus, in the current study, we investigated the beneficial effects of herbal prescription, PMC-12, on hippocampal neurons and spatial memory deficits in mice.

Exposure to glutamate causes neuronal cell death via non-receptor-mediated oxidative stress in hippocampal HT22 cells [28, 29]. Increased levels of Ca^2+^ and ROS caused by exposure to glutamate lead to both apoptotic and necrotic cell death [6, 30, 31]. Since alteration of Ca^2+^ signaling is involved in cell death, it can increase ROS production [32–34].

In the current study, treatment with PMC-12 resulted in reduction of apoptotic cell death with suppression of ROS accumulation; PMC-12 may contribute to neuronal survival against glutamate-induced oxidative toxicity related to cellular content of ROS and Ca^2+^. However, in treatment with PMC-12, ROS production was not significantly diminished in a concentration-dependent manner and cells were effectively protected for an additional 24 after removal of PMC-12. The beneficial effect of PMC-12 on hippocampal cells may not be attributable to its direct control of ROS accumulation and Ca^2+^ influx. With anti-oxidative activities, PMC-12 may contribute to other protective pathways, including endogenous kinases.

Neuroprotective effects under oxidative stress are mediated by regulation of MAPK and PI3K signaling pathways, leading to cell death or survival in a variety of neurodegenerative disorders [35–37]. In particular, roles of p38 MAPK signaling in neuronal death [38, 39] and activation of PI3K in neuroprotection [10, 37] play an essential role under oxidative stress conditions. Our results showed that neuroprotective effects of PMC-12 are regulated by signaling pathways of p38 MAPK and PI3K against glutamate-induced oxidative cell death.

CREB, an important transcription factor implicated in control of adaptive neuronal responses, contributes to several critical functions of BDNF-mediated cell survival [40, 41]. The impaired CREB phosphorylation in hippocampus may be a pathological component in neurodegenerative disorders [42, 43]. BDNF has recently been recognized as a potent modulator capable of regulating a wide repertoire of neuronal functions [44]. Among two forms of extracellular BDNF, pro- and mature BDNF, mature BDNF is essential in protection of neonatal or developing brain from ischemia injury as well as neuronal cells, whereas pro-BDNF may induce neuronal death [44–48]. In accordance with previous studies, cells treated with PMC-12 recovered protein levels of mature BDNF and CREB phosphorylation caused by glutamate. These results suggest that neuroprotective effects of PMC-12 may be regulated by both mature BDNF expression and CREB phosphorylation.

Memory improvement is indicative of the structural and functional hippocampal plastic changes including its expression of BDNF [22]. To confirm involvement of mature BDNF and CREB activation in neuroprotection of PMC-12, we performed *in vivo* study using a mild ischemic mouse model [22, 23]. Immunohistochemistry results of treatment with PMC-12 for three weeks in MCAO mice indicated significant expression in the CA1 and DG regions of the hippocampus, suggesting that PMC-12 may prevent neuronal death via signaling pathways of both mature BDNF expression and CREB phosphorylation.

In addition, we applied a behavioral test, Morris water maze, which assesses MCAO-induced deficits of learning and memory. Previous report showed that a short duration of 60 min MCAO instead of 2 h does not result in concomitant sensorimotor deficits in mice [23, 49]. Thus 30 min MCAO was conducted to avoid the influence of sensorimotor deficit on the performance of the mice in our Morris water maze test. Treatment with PMC-12 resulted in attainment of a significantly lower time from 22 to 25 days after MCAO compared with the vehicle group. These findings suggest that PMC-12 may be a good candidate for recovery of damaged learning and memory function in a focal cerebral ischemia model.

Our study did not investigate the main functional components of multiherb formula PMC-12. However, phenolic constituents are well known as major active components of *Polygonum multiflorum* [50] and tetrahydroxystilbene-glucoside identified from this herb promotes long-term potentiation inductions contributing to enhancement of learning and memory in mice models [51, 52]. In line with these studies, our HPLC analysis also showed that 2,3,5,4′-tetrahydroxystilbene-2-O-β-D-glucoside is one of the main components of multiherb formula PMC-12. However, medical herbs have diverse roles within multiherb formulae according to well-known perspectives of traditional Korean medicine. Some herbs target the primary disorder and others aim to alleviate secondary symptoms such as absorption or counting undesirable effects of other herbs [24, 53]. PMC-12 may enhance memory function to target primary neuroprotective effects and other secondary symptoms by diverse roles within multiherb formulae.

## Conclusions

PMC-12 protects against hippocampal neuronal cell death via inhibition of Ca^2+^-related accumulation of ROS and these effects regulate through the signaling pathways of p38 MAPK and PI3K associated with mature BDNF expression and CREB phosphorylation. PMC-12 may also modulate recovery of memory function via mature BDNF and CREB against cerebral ischemic stroke. These results have shown that multiherb formula PMC-12 has potential applications as a useful therapeutic strategy in memory impairment of brain disorders.
