# Erratum to: Neuroprotection and spatial memory enhancement of four herbal mixture extract in HT22 hippocampal cells and a mouse model of focal cerebral ischemia

**DOI:** 10.1186/s12906-015-0796-z

**Published:** 2015-08-13

**Authors:** Sung Min Ahn, Yu Ri Kim, Ha Neui Kim, Young Whan Choi, Jae Won Lee, Cheol Min Kim, Jin Ung Baek, Hwa Kyoung Shin, Byung Tae Choi

**Affiliations:** Department of Korean Medical Science, School of Korean Medicine, Pusan National University, Yangsan, 626-870 Republic of Korea; Department of Horticultural Bioscience, College of Natural Resource and Life Science, Pusan National University, Miryang, 626-706 Republic of Korea; Division of Pharmacy, College of Pharmacy, Pusan National University, Busan, 609-735 Republic of Korea; Department of Biochemistry, College of Medicine, Pusan National University, Yangsan, 626-870 Republic of Korea; Division of Humanities and Social Medicine, School of Korean Medicine, Pusan National University, Yangsan, 626-870 Republic of Korea; Division of Meridian and Structural Medicine, School of Korean Medicine, Pusan National University, Yangsan, 626-870 Republic of Korea; Research Center for Anti-aging Technology Development, Pusan National University, Busan, 609-735 Republic of Korea

Unfortunately, the original version of this article [1] contained an error. The corrected Figure four (Fig. 1) can be found below. The Figure four (Fig. 1) has been corrected in the original article and is also included correctly below.

**Fig. 1:**
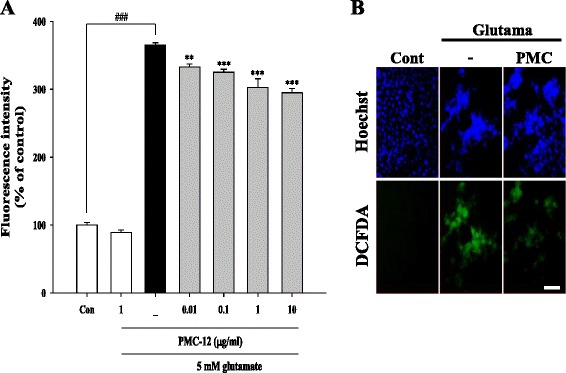
Protective effect of PMC-12 on ROS generation in glutamate-treated HT22 cells. Cells were pretreated with 0.01, 0.1, 1, or 10 μg/ml of PMC-12 for 24 h, followed by exposure to 5 mM glutamate for 24 h. The oxidation sensitive fluorescence dye, carboxy-H _2_ DCFDA (20 μM), was used in measurement of ROS levels. Production of ROS was analyzed using a fluorescence plate reader (**a**) and fluorescence microscope (**b**). In addition, apoptotic nuclei were observed after staining with Hoechst 33342 for detection of apoptosis morphologically (**b**). ^###^ *P* < 0.001 vs. control; **P < 0.01 and ***P < 0.001 vs. glutamate-treated cells. All data are represented as the mean ± SEM of three independent experiments. Scale bars = 50 μm
